# Atmospheric Behaviour of Polycyclic and Nitro-Polycyclic Aromatic Hydrocarbons and Water-Soluble Inorganic Ions in Winter in Kirishima, a Typical Japanese Commercial City

**DOI:** 10.3390/ijerph18020688

**Published:** 2021-01-14

**Authors:** Lu Yang, Quanyu Zhou, Hao Zhang, Xuan Zhang, Wanli Xing, Yan Wang, Pengchu Bai, Masahito Yamauchi, Tetsuji Chohji, Lulu Zhang, Kazuichi Hayakawa, Akira Toriba, Ning Tang

**Affiliations:** 1 Graduate School of Medical Sciences, Kanazawa University, Kakuma-machi, Kanazawa 920-1192, Japan; veronicayl@stu.kanazawa-u.ac.jp (L.Y.); zhouquanyu1995@gmail.com (Q.Z.); zhanghao@stu.kanazawa-u.ac.jp (H.Z.); zhangxuan@stu.kanazawa-u.ac.jp (X.Z.); xingwanli@stu.kanazawa-u.ac.jp (W.X.); wangyan@stu.kanazawa-u.ac.jp (Y.W.); baipengchu@stu.kanazawa-u.ac.jp (P.B.); 2 National Institute of Technology, Kagoshima College, Hayatocho, Kirishima 899-5193, Japan; yamauti@kagoshima-ct.ac.jp (M.Y.); chohji@kagoshima-ct.ac.jp (T.C.); 3 Institute of Nature and Environmental Technology, Kanazawa University, Kakuma-machi, Kanazawa 920-1192, Japan; zhang-lulu@se.kanazawa-u.ac.jp (L.Z.); hayakawa@p.kanazawa-u.ac.jp (K.H.); 4 School of Pharmaceutical Sciences, Nagasaki University, Bunkyo-machi, Nagasaki 852-8521, Japan; toriba@nagasaki-u.ac.jp; 5 Institute of Medical, Pharmaceutical and Health Sciences, Kanazawa University, Kakuma-machi, Kanazawa 920-1192, Japan

**Keywords:** air pollution, polycyclic aromatic hydrocarbon, nitro-polycyclic aromatic hydrocarbon, water-soluble inorganic ions, Kirishima

## Abstract

Kirishima is a typical Japanese commercial city, famous for frequent volcanic activity. This is the first study to determine the characteristics of PM_2.5_-bound polycyclic and nitro-polycyclic aromatic hydrocarbons (PAHs and NPAHs) and water-soluble inorganic ions (WSIIs) in this city. In this study, the non-volcanic eruption period was taken as the target and daily PM_2.5_ samples were collected from 24 November to 21 December 2016. The daily concentrations in PM_2.5_ of ƩPAHs, ƩNPAHs, and ƩWSIIs ranged from 0.36 to 2.90 ng/m^3^, 2.12 to 22.3 pg/m^3^, and 1.96 to 11.4 μg/m^3^, respectively. Through the results of the diagnostic ratio analyses of the PAHs, NPAHs, and WSIIs and the backward trajectory analysis of the air masses arriving in Kirishima, the emission sources of PAHs, NPAHs, and WSIIs in PM_2.5_ in Kirishima were influenced by the coal burning that came from the East Asian continent, although there was no influence from volcanic emission sources during the sampling period. The total benzo[*a*]pyrene (BaP)-equivalent concentration was lower than many other cities but the health risks in Kirishima were nonetheless notable. These findings are very important for future research on PM samples during the inactive Asian monsoon and volcanic eruption periods, to further understand the characteristics of air pollutants in Kirishima, and to contribute to the improvement in health of residents and a reduction in the atmospheric circulation of air pollutants in East Asia.

## 1. Introduction

Many studies have highlighted that atmospheric particulate matter (PM) is closely related to human health [1,2,3]. The potential toxicity of PM depends on its size and chemical composition [1,4,5]. Regarding the size of PM, fine particles (PM_2.5_) penetrate the respiratory system more easily than coarse particles and become deposited deeply in the alveoli, causing various diseases in humans [6,7]. Regarding the chemical composition of PM, polycyclic aromatic hydrocarbons (PAHs) and their nitrated derivatives (NPAHs) are organic compounds in PM that are well known for their carcinogenicity and mutagenicity [8,9,10]. The International Agency for Research on Cancer (IARC) has classified dozens of PAHs and NPAHs as carcinogenic to humans (Groups 1, 2A, and 2B) [11,12,13]. Meanwhile, PAHs have been reported associated with adult chronic cough [14]. Water-soluble inorganic ions (WSIIs) are the main inorganic compounds in PM [15]; these ions can change the size and composition of PM, thereby changing or even increasing the toxicity of PM [16,17]. Moreover, WSIIs can alter atmospheric acidification and the acidity of cloud mist, which can promote toxic and harmful substances dissolved into the human body [18]. PAHs and most NPAHs in the atmosphere originate mainly from the incomplete burning of organic materials, such as fossil fuel and biomass [19,20,21], while several NPAHs are generated only through atmospheric reactions, such as 2-nitropyrene (2-NP) and 2-nitrofluoranthene (2-NFR) [22]. WSIIs not only exist widely in natural sources such as dust and sea salt but can also originate from primary combustion processes and secondary atmospheric reactions [23,24].

Kirishima city is located in southern Kyushu, Japan, and is a typical commercial city and the second-most populous city in Kagoshima Prefecture. There are several highly active volcanoes in the vicinity of the city, including Mt. Sakurajima, an active volcano located across Kagoshima Bay, and Mt. Kirishima, an active volcano group located between Kagoshima and Miyazaki Prefectures. Volcanoes are a well-known natural source of volcanic ash and gases that create haze, alter the composition of the atmosphere, and significantly affect the climate [25]. Due to the urban characteristics and specific geographical location of Kirishima, air pollutants in the city can come from both anthropogenic sources and natural sources, such as automobiles and volcano activity, respectively. Moreover, because Japan is in the range of the leeward wind of the East Asian winter monsoon, air pollutants in Kirishima may be long-range transported from the Asian continent in cold periods [26,27]. However, because of the specific geographical environmental context of Kirishima, research on air pollution in Kirishima has so far focused mainly on the health and ecosystem impacts during volcanic activity. For example, Shinohara [28] reported the volcanic gas composition during the eruption of Shinmoedake, one of Kirishima’s volcanos, whereas the atmospheric behaviour of PM and its components during periods without volcanic activity have been overlooked. Urban background research on PM is also important for human health and regional climate. To our best knowledge, PAHs and NPAHs in PM have not been reported in Kirishima.

Therefore, this study specifically chose to collect PM_2.5_ samples during an East Asian winter monsoon period without volcanic activity. Nine PAHs, three NPAHs, and eight WSIIs in the PM_2.5_ were analysed. Through diagnostic ratio analysis, WSII acid-base balance analysis, and backward trajectory analysis, the concentrations, compositions, and potential emission sources of these species were clarified, especially the influence of long-range transport from the Asian continent. This study was intended to reveal the specific characteristics and the human health risks of air pollution in Kirishima.

## 2. Materials and Methods

### 2.1. PM_2.5_ Sampling

**Figure 1:**
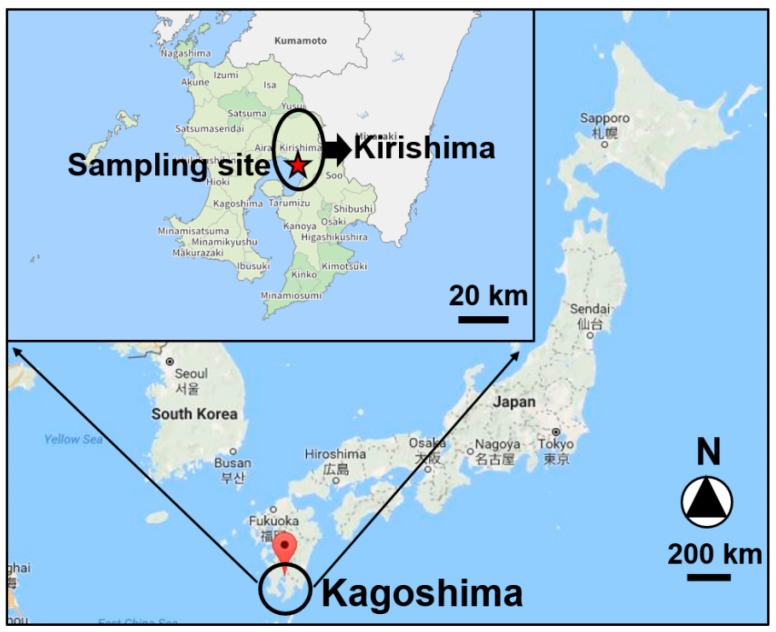
Location of sampling site (National Institute of Technology, Kagoshima, 31°43′48″ N, 130°43′12″ E).

As shown in Figure 1, PM_2.5_ sampling was performed at the National Institute of Technology, Kagoshima College (31°43′48″ N, 130°43′12″ E), which is located in Kirishima city, Kagoshima Prefecture, Japan. The air sampler was set up on the rooftop of a building 5 m above the ground and approximately 500 m from the road. Daily PM_2.5_ samples were collected from 24 November to 21 December 2016, using a high-volume air sampler (HV-1000F, Sibata Scientific Technology Ltd., Sibata, Japan) equipped with quartz fiber filters (2500QAT-UP, 8 × 10 inches, Pall Co., Port Washington, NY, USA) at a flow rate of 1000 L/min. The sampling started at 16:30 and continued for 24 h, and the filters were changed the next day at 16:30. After sampling, PM_2.5_ sample filters were packaged in aluminum foil, sealed in plastic bags, and stored at −25 °C until experimental analysis.

### 2.2. PAH, NPAH, and WSII Analyses and Chemical Compositions

Descriptions of the PAH and NPAH pretreatment methods can be found in our previous study [29]. The PM_2.5_ samples were cut into small pieces and combined with internal standards (pyrene-*d*_10_ (Pyr-*d*_10_), benzo[*a*]pyrene-*d*_12_ (BaP-*d*_12_), and 2-fluoro-7-nitrofluorene (FNF)) in flasks. After two ultrasonic extractions by adding benzene:ethanol (3:1, *v*/*v*), the extracted solution was washed successively with sodium hydroxide solution (5%; *w*/*v*), sulfuric acid solution (20%; *v*/*v*), and water. Then, the solution was concentrated to 100 µL, and ethanol was added to bring the concentrated residue up to 1 mL. After pretreatment, nine PAHs and three NPAHs were separately detected by a high-performance liquid chromatography (HPLC) fluorescence detection system [30]. The WSIIs in PM_2.5_ samples were ultrasonically extracted in ultrapure water. The extracted solution was divided into two parts, and eight WSIIs, including five cations and three anions, were separately detected by the ion chromatography (IC) system [14].

PAH, NPAH, and WSII standard solutions were injected into the HPLC and IC systems to check the HPLC and IC analysis methods before actual sample detection. PAH, NPAH, and WSII standard solutions with different concentration gradients were tested at least three times. The relative standard deviations of all species were within 5%. The calibration curves of all PAHs, NPAHs, and WSIIs exhibited good linearity (*r* > 0.998). Three blank filters were also analysed under the same pretreatment as the samples. The analyses of the blank filters showed that none of the target chemicals were detected, indicating that there was no background contamination during the transport process. The recoveries of the internal standards were used to calibrate the concentrations and to determine whether errors occurred during the experiment. Pyr-*d*_10_ was used for the 4-ring PAHs (fluoranthene (FR), pyrene (Pyr), benz[*a*]anthracene (BaA), and chrysene (Chr)), and BaP-*d*_12_ was used for the 5-ring PAHs (benzo[*b*]fluoranthene (BbF), benzo[*k*]fluoranthene (BkF), and benzo[*a*]pyrene (BaP)) and 6-ring PAHs (benzo[*ghi*]perylene (BgPe) and indeno[1,2,3-cd]pyrene (IDP)). FNF was used for the three NPAHs (1-NP, 2-NP, and 2-NFR). The recoveries of internal standards in all samples in this study were within 20%. Table 1 shows the name and abbreviation of the target species of PAHs, NPAHs, and WSIIs. Table S1 (Supplementary Materials) lists the limit of determination (LOD) of each PAH, NPAH, and WSII species.

In this study, the PAH standard solution (US EPA 610 PAH mix) was purchased from Supelco Park (Bellefonte, PA, USA); 1-nitropyrene (1-NP), 2-NP, and FNF were purchased from Aldrich Chemical Company (Osaka, Japan); 2-NFR was purchased from Chiron AS (Trondheim, Norway); and Pyr-*d*_10_, BaP-*d*_12_, and WSII standard solutions were purchased from Wako Pure Chemicals (Osaka, Japan). All other analytical reagent-grade reagents used in the HPLC and IC analyses were purchased from Wako Pure Chemicals (Osaka, Japan).

### 2.3. Data Analysis

#### 2.3.1. Meteorological Conditions

Meteorological data, including the average temperature, precipitation, relative humidity, sunshine hours, wind speed, and prevailing wind direction in Kirishima during the sampling period shown in Table S2 (Supplementary Materials), were obtained from the Japan Meteorological Agency (http://www.jma.go.jp/jma/menu/menureport.html).

#### 2.3.2. Cation Equivalent (CE), Anion Equivalent (AE), and Non-Sea Salt (nss-) WSIIs

The WSII acid-base balance at Kirishima during the sampling period was analysed by using CE and AE, which were calculated as follows [31]:

AE = [SO_4_^2−^]/48 + [NO_3_^−^]/62 + [Cl^−^]/35.5 (1)

CE = [NH_4_^+^]/18 + [Mg^2+^]/12.2 + [Ca^2+^]/20 + [K^+^]/39 + [Na^+^]/23 (2)

The concentrations of nss-SO_4_^2−^, nss-K^+^, nss-Ca^2+^, and nss-Mg^2+^ were calculated as follows [24]:

[nss-SO_4_^2−^] = [SO_4_^2−^] − [Na^+^] × 0.2516 (3)

[nss-K^+^] = [K^+^] − [Na^+^] × 0.037 (4)

[nss-Ca^2+^] = [Ca^2+^] − [Na^+^] × 0.038 (5)

[nss-Mg^2+^] = [Mg^2+^] − [Na^+^] × 0.12 (6)

where [SO_4_^2−^], [NO_3_^−^], [Cl^−^], [NH_4_^+^], [Mg^2+^], [Ca^2+^], [K^+^], and [Na^+^] are the concentrations.

#### 2.3.3. Backward Trajectory

The air masses that flowed into Kirishima during the sampling period were analysed by their backward trajectories, which were downloaded from the U.S. National Oceanic and Atmospheric Association’s HYSPLIT4 model (WINDOWS-based). In this study, each backward trajectory was calculated every hour at a sampling point height of 500 m above ground level, with a tracking time of 72 h. All backward trajectories during the sampling period were classified into four clusters according to their characteristics.

#### 2.3.4. Health Risk Assessment

The BaP-equivalent (BaP_eq_) concentrations were expressed as follows:

BaP_eq_ = ∑(C*_i_* × TEF*_i_*) (7)

where C*_i_* is the concentration of each species (pg/m^3^) and TEF*_i_* is the toxic equivalency factor of each species relative to BaP, obtained by referring to previous studies [8,9,32]. The inhalation lifetime cancer risk (ILCR) from exposure to PAHs and NPAHs was expressed as follows:

ILCR = UR_BaP_ × BaP_eq_ (8)

where UR_BaP_ is the unit cancer risk from BaP, which was assigned a value of 8.7 × 10^−5^ per ng/m^3^ in this study [33].

#### 2.3.5. Statistical Analysis

Statistical analysis of the data was performed using IMB SPSS version 25.0. Spearman correlation analysis was used to determine the correlation between PAHs, NPAHs, and WSIIs. Differences in the results were considered significant at *p* values of less than 0.05 or 0.01.

## 3. Results and Discussion

### 3.1. Concentrations of PAHs, NPAHs, and WSIIs

Table 1 summarizes the concentrations of PAHs, NPAHs, and WSIIs in Kirishima during the sampling period. The daily concentrations of ƩPAHs ranged from 0.36 to 2.90 ng/m^3^, with an average of 1.32 ± 0.71 ng/m^3^; this level is comparable to those from other Japanese commercial cities such as Sapporo (1.79 ng/m^3^) and Sagamihara (1.83 ng/m^3^) in winter 2013 [34] and Kanazawa (1.00 ng/m^3^) in winter 2018 [35], but lower than those from other Asian cities such as Shanghai, China in winter 2018 (7.72 ng/m^3^) [30], Beijing, China in winter 2015 (264 ng/m^3^) [36], Shenyang, China in winter from 2012 to 2014 (65.7–244 ng/m^3^) [37], and Ulaanbaatar, Mongolia in winter 2017 (131–773 ng/m^3^) [38]. The daily concentrations of ƩNPAHs ranged from 2.12 to 22.3 pg/m^3^, with an average of 9.98 ± 5.75 pg/m^3^. The concentration level of ƩNPAHs was much lower than that of ƩPAHs in this study, which is also consistent with results from the urban cities listed above [30,34,35,36].

The daily concentrations of ƩWSIIs ranged from 1.96 to 11.4 μg/m^3^, with an average of 5.74 ± 2.59 μg/m^3^; this level is slightly lower than those of other cities of the same type in Japan, such as Osaka in 2015 (8.1 μg/m^3^) [39] and Yokohama from 1999 to 2005 (9.83 μg/m^3^) [40], and much lower than those of other Asian cities, such as Zhengzhou, China in 2014 (83.7 μg/m^3^) [41], Ningbo, China in 2015 (25.5 μg/m^3^) [24], Changzhou, China in 2016 (66.8 μg/m^3^) [42], and Ulaanbaatar, Mongolia in 2017 (23.2 μg/m^3^) [43]. The Spearman correlation analysis showed that there were strong positive correlations among PAHs, NPAHs, and WSIIs (*p* < 0.01), indicating that there were some internal connections between these species, although the main sources of emissions of these species were not entirely the same.

### 3.2. Composition of PAHs, NPAHs, and WSIIs

As shown in Table 1, FR had the highest average concentration (0.31 ± 0.20 ng/m^3^) during the sampling period. The average concentrations of Pyr, BbF, BgPe, and IDP ranged from 0.16 to 0.18 ng/m^3^, higher than those of the other PAHs. The average proportions of 4-, 5- and 6-ring PAHs during the sampling period accounted for approximately 47.0%, 25.7%, and 27.4% of the ƩPAHs, respectively. Four-ring PAHs that originated mainly from coal and biomass burning made up a relatively large proportion of the ƩPAHs, which is consistent with other reports [44,45,46]. This phenomenon occurs because 4-ring PAHs can be transferred from the gaseous phase to the particle phase easily at low ambient temperatures in winter due to vapor pressure [47] and may also be related to the emission sources that will be discussed in Section 3.3. Among the three NPAHs, 2-NFR had the highest average concentration (7.75 ± 4.59 pg/m^3^), making up 72% to 84% of the daily ƩNPAHs, followed by 1-NP (1.77 ± 1.02 pg/m^3^), which constituted 11% to 21% of the daily ƩNPAHs during the sampling period (Table 1). Additionally, consistent with previous studies, the concentration of 2-NFR was higher than those of 1-NP and 2-NP; of these NPAH types, 2-NFR and 2-NP are secondarily generated [30,48,49].

**Figure 2:**
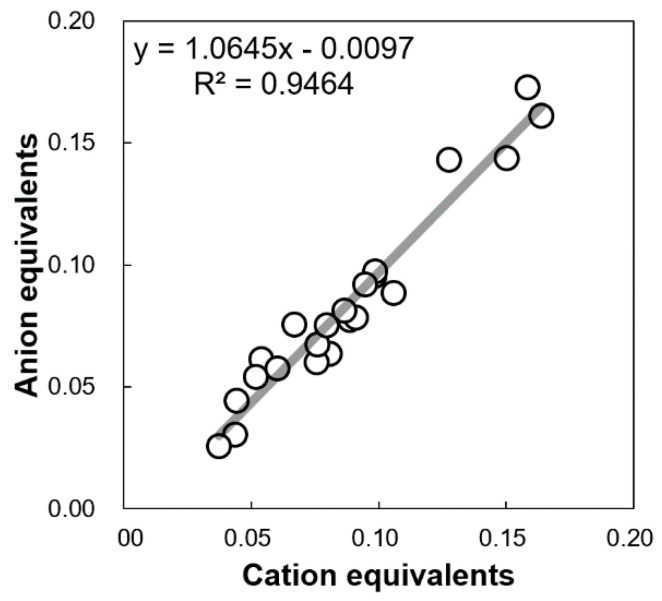
WSIIs acid-base balance at Kirishima during the sampling period.

Among the eight WSIIs, SO_4_^2−^ had the highest average concentration (3.78 ± 1.77 μg/m^3^), followed by NH_4_^+^ (1.35 ± 0.59 μg/m^3^). Moreover, the concentration of NO_3_^−^ (0.28 ± 0.23 μg/m^3^) was also higher than those of the other WSIIs (Table 1). These three species constituted at least 85% of the daily ƩWSIIs and are therefore the main WSII species of PM_2.5_, which is consistent with previous studies [24,41,50]. Because anions can increase the acidity of PM and cations can increase the alkalinity of PM, the AE/CE ratio is a good indicator for determining the acidity or alkalinity of PM [23]. As shown in Figure 2, AE/CE was 1.06, with good linearity (*r* = 0.97), indicating that PM_2.5_ was relatively neutral at Kirishima during the sampling period. Moreover, the AE/CE value, which was close to 1, also corroborates the validity of the WSII measurements, indicating that most WSII species were quantified [51].

### 3.3. Potential Emission Sources

**Figure 3:**
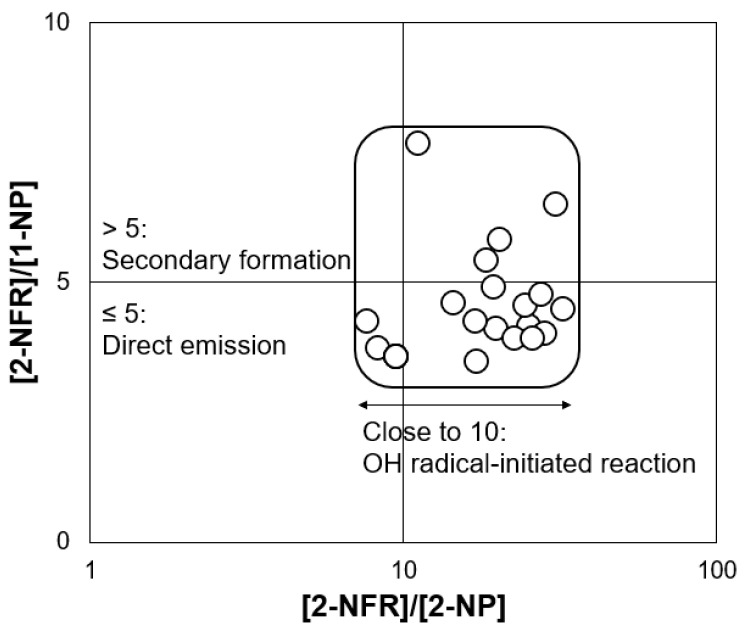
Ratios of [2-NFR]/[1-NP] and [2-NFR]/[2-NP] at Kirishima during the sampling period.

Table 2 summarizes some of the diagnostic ratios of PAHs, NPAHs, and WSIIs at Kirishima during the sampling period. The [FR]/([FR] + [Pyr]) ratios ranged from 0.50 to 0.74, with an average of 0.61, and the [BaA]/([BaA] + [Chr]) ratios ranged from 0.34 to 0.52, with an average of 0.41. Compared to the reference value of PAH diagnostic ratios emitted from coal burning and traffic emissions [52,53], the potential source of the emissions in this study was coal burning. Moreover, the [1-NP]/[Pyr] ratios, which ranged from 0.005 to 0.019 and had an average of 0.008, was also close to that of coal burning emissions [21]. However, the [BbF]/([BbF] + [BkF]) ratios ranged from 0.72 to 0.76, the [BaP]/[BgPe] ratio ranged from 0.32 to 0.90, and the [IDP]/([IDP] + [BgPe]) ratios ranged from 0.34 to 0.58; these values are between those for coal burning and traffic emissions, indicating mixed sources for the PAHs in this study [37,53,54]. Of the three NPAHs, 1-NP is primarily formed, and 2-NFR and 2-NP are secondarily formed [48]. As shown in Figure 3, the [2-NFR]/[1-NP] ratios ranged from 3.50 to 7.68, with an average of 4.51, and its values were mostly lower than 5. These values indicate the higher contribution of direct emissions such as coal burning during the sampling period [55]. The [NO_3_^−^]/[SO_4_^2−^] ratio is usually used to estimate the relative importance of traffic emissions and coal burning sources [42]. Table 2 shows that these ratios ranged from 0 (NO_3_^−^ concentration was less than the LOD) to 0.32, with an average of 0.11. Ratios lower than 1.0 indicate that the emission sources were more likely related to coal burning [42]. Consequently, the diagnostic ratios of PAHs, NPAHs, and WSIIs indicated that the main sources at Kirishima during the sampling period were mixed but that coal burning made a higher contribution than traffic emissions.

On the other hand, Figure 3 also shows that the [2-NFR]/[2-NP] ratios ranged from 7.61 to 32.0, with an average of 18.4. Values of this ratio near 10 indicate that 2-NFR is mainly secondarily formed by the OH radical-initiated reaction rather than formed through the NO_3_ radical-initiated reaction, which was similar to the results for 2-NP [55]. Moreover, some WSII species had both sea-salt sources and non-sea-salt sources. According to the calculations from Equations (3)–(5), the concentrations of [nss-SO_4_^2−^], [nss-K^+^], and [nss-Ca^2+^] accounted for 90% to 99% of the total SO_4_^2−^, K^+^, and Ca^2+^, indicating that these species were mostly emitted from non-sea-salt sources. Table 3 shows the concentration ratios of these species to Na^+^ during the sampling period. According to the reference data [56], the ratios of [SO_4_^2−^]/[Na^+^], [K^+^]/[Na^+^], and [Ca^2+^]/[Na^+^] all suggested that they were more abundant in PM_2.5_ than in sea salt. However, the [nss-Mg^2+^]/[Mg^2+^] percentages ranged from 0% to 82%, with an average of 47%, and the [Mg^2+^]/[Na^+^] ratios ranged from 0.10 to 0.66, with an average of 0.26 (Table 3); both of these results suggest that sea salt had a relatively large impact as a source of Mg^2+^ [56].

### 3.4. Backward Trajectory Analysis

**Figure 4:**
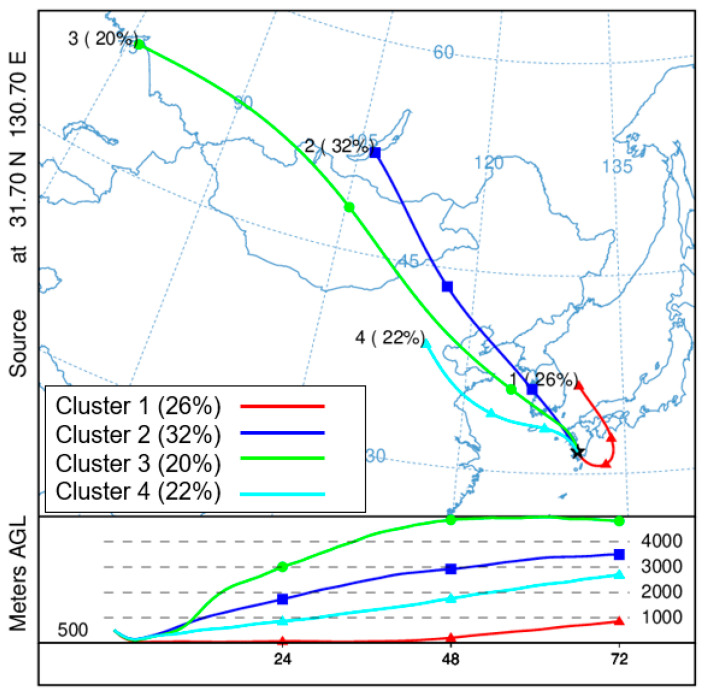
Cluster analysis of 72-h tracking backward trajectories at Kirishima during the sampling period.

Figure 4 shows the source areas of the air masses that arrived at Kirishima during the sampling period determined by performing a cluster analysis of their tracked 72-h backward trajectories. Of the four clusters, clusters 2, 3, and 4 constituted approximately 74% of all the air masses and all came from the northwest direction, consistent with the prevailing wind direction at Kirishima (NNW; Table S2, Supplementary Materials). Specifically, cluster 1 contained 26% of the air masses, which came from the Sea of Japan and then moved across domestic Japan and from the Pacific Ocean to Kirishima. Clusters 2 and 3 contained 32% and 20% of the air masses, respectively, which came from different source areas in Russia and moved across both Mongolia and North China. Cluster 4 contained 22% of the air masses, which came from North China and then passed across the Yellow Sea to Kirishima. These results were consistent with previous studies showing that the air masses that arrived in Japan in the wintertime came mostly from the Asian continent [57,58]. The source areas of Mongolia and northern China, which the air masses came from or passed through, contained high concentrations of air pollutants during the sampling period because biomass burning for warmth is common in Mongolia [43] and coal burning in heating systems is common in North China [46]. In addition, Figure 4 shows that the height ranges of the air masses that came from local Japan and the ocean (cluster 1) were lower than 1000 m, which is much lower than those that came from the Asian continent, including Russia, Mongolia, and China (clusters 2, 3, and 4). These results suggest that the air pollutants originating from emission areas in Japan and the sea are very likely to sink during the long-range transport process [26] and thus are less likely to arrive at Kirishima than those originating from the Asian continent. The diagnostic ratios discussed in Section 3.3 suggested that coal burning had a larger impact than other sources and that the direct emission of NPAHs made a high contribution. These results may be because the air masses from the Asian continent arriving at Kirishima contained these species emitted from combustion sources that did not undergo substantial degradation during the long-range transport process; this phenomenon has also been reported in previous studies [26,27,48].

According to the daily concentrations of each species shown in Tables S3 and S4 (Supplementary Materials), the concentrations of ∑PAHs and ∑NPAHs on 26 November 2016 (∑PAHs: 364 pg/m^3^; ∑NPAHs: 2.12 pg/m^3^) and 12 December 2016 (∑PAHs: 382 pg/m^3^; ∑NPAHs: 2.89 pg/m^3^) were lower than those on other days. As shown in Table S5 (Supplementary Materials), the main source areas for the air masses on these two days were both domestic across the Pacific Ocean and Kirishima, and the height ranges of the air masses were both lower than 500 m; these air masses were in cluster 1. Previous studies have reported that air masses coming from or passing through the ocean contain a relatively low concentration of air pollutants because the sea has a diluting effect on air pollutants [27,29]. Moreover, the meteorological conditions shown in Table S2 (Supplementary Materials) showed that the precipitation was higher on 27 November 2016 (29.5 mm) and 13 December 2016 (46.5 mm). These results suggest that the rain-out effect had a positive effect on cleaning PM suspended in the atmosphere [59], leading to low concentrations of ∑PAHs and ∑NPAHs on 26 November and 12 December 2016 because the filters for those two days were changed at 16:30 on 27 November and 13 December 2016. On the other hand, although the concentration of WSIIs on 12 December 2016 was also low, the concentration on 26 November 2016 was closer to the average concentration (Table S5, Supplementary Materials). Table S2 (Supplementary Materials) indicates that the prevailing wind direction on 26 November 2016 was NNW, which was different from that on 12 December 2016 (NNE), suggesting that the ground source areas were different. This difference may have led to the difference in WSII concentrations between these two days because WSIIs can not only be emitted from combustion sources but can also come from non-combustion sources such as road dust [31,56].

As shown in Table 1, the median concentrations of most PAHs, NPAHs, and WSIIs were lower than their average concentrations. In particular, the median concentration of ∑PAHs was 18.9% lower than the average level, suggesting that high concentrations had relatively large impacts on the total concentration during the whole period. According to the daily concentrations shown in Table S3 (Supplementary Materials), there were nine days, including 25 and 27 to 29 November and 1, 5, 6, 8, and 9 December 2016, on which the concentrations of ∑PAHs were higher than the average level (1.32 ng/m^3^); the daily concentrations of NPAHs on those nine days were also higher than the average level (9.98 pg/m^3^). In addition, there were six days among these nine days in which the daily concentrations of ∑WSIIs were higher than the average level (5.74 µg/m^3^). Table S5 (Supplementary Materials) shows that all air masses on these days came from the Asian continent, with high height ranges, except those on 29 November and 8 December 2016, which had relatively low heights. This suggests that the air masses arriving at Kirishima contained relatively high concentrations of air pollutants [21]. Therefore, the impact of air masses from the Asian continent in winter on Kirishima cannot be ignored, although not all air masses from the Asian continent showed high concentrations of PAHs, NPAHs, and WSIIs.

### 3.5. Health Risk Assessment

Table 4 summarizes the BaP_eq_ concentrations of nine PAHs, 1-NP, and 2-NFR (2-NP had no available TEF value) at Kirishima during the sampling period. The ƩBaP_eq_ concentrations ranged from 31.2 to 302 pg/m^3^, with an average of 142 pg/m^3^, and the nine PAHs contributed mostly to ƩBaP_eq_ concentrations. In addition to BaP (90.9 pg/m^3^), BbF (18.7 pg/m^3^) and IDP (16.3 pg/m^3^) had the highest BaP_eq_ concentrations, indicating that they represented higher health risks than the other species. On the other hand, although the 1-NP and 2-NFR concentrations were much lower than those of the nine PAHs (Table 1), the BaP_eq_ concentrations of 1-NP (0.18 pg/m^3^) and 2-NFR (0.39 pg/m^3^) were comparable to those of FR (0.31 pg/m^3^) and Pyr (0.18 pg/m^3^) because the TEF values of 1-NP and 2-NFR were higher than those of FR and Pyr (Table 4) [8,9,32].

The ILCR at Kirishima during the sampling period was 1.22 × 10^−5^, indicating that approximately 12 cancer cases may occur among 10^6^ people due to PAHs and NPAHs exposure. The ILCR in this study was much lower than that in other Asian cities that used the same UR_BaP_ value [30,37,60]. However, it was one order of magnitude over the acceptable level established by the US EPA (10^−6^), indicating that exposure to PAHs and NPAHs at the levels observed in this study has adverse effects on human health. On the other hand, the UR_BaP_ value used to calculate the ILCR in this study was obtained from an epidemiological study of coke oven workers whose ILCR was very high [33]; this created some uncertainty in determining the risk of exposure to PAHs and NPAHs for the non-professional population.

## 4. Conclusions

The characteristics of PM_2.5_-bound PAHs, NPAHs, and WSIIs in Kirishima, Japan, in an urban context without specific natural activity were investigated in this study. The concentrations of PM_2.5_-bound PAHs, NPAHs, and WSIIs at Kirishima were comparable to those in other Japanese cities and lower than those in many other Asian cities in a similar period. Meteorological conditions such as precipitation can have a strong impact on the concentrations of air pollutants. The air masses that arrived at Kirishima came mostly from the Asian continent and may have contained high levels of air pollutants emitted from coal burning. Sea salt had a larger impact on Mg^2+^ than on other WSII species. Moreover, PAHs contributed the majority of the ƩBaP_eq_ concentration, and the main contributors to the ƩBaP_eq_ concentration in this study were BaP, BbF, and IDP; however, the health risks of NPAHs could not be ignored.

Kirishima is one of the typical commercial cities in the Kyushu area, Japan. The emission source of PAHs was not complex, and the urban background concentration was low. This study found that the air pollutants in Kirishima were also influenced by the air masses long-range transported from the East Asian continent during the East Asian winter monsoon period, similar to the region of the Sea of Japan. This finding is not only a reminder for the operation of environmental protection policies, but it can also be a reminder for other similar areas. Although the PM_2.5_ samples were only collected at one site, this study is the first to determine the atmospheric behaviour of PAHs, NPAHs, and WSIIs in PM_2.5_ in Kirishima, leading to a basic understanding. In the future research, we need to collect PM_2.5_ samples simultaneously at different sites in Kirishima in other seasons and specific periods of natural activity such as volcanic eruptions, and to analyse other pollutants such as gaseous pollutants, to further determine their atmospheric behaviours in Kirishima.

## Figures and Tables

**Table 1:** Abbreviation and concentrations of polycyclic aromatic hydrocarbons (PAHs), nitro-polycyclic hydrocarbons (NPAHs), and water-soluble inorganic ions (WSIIs) at Kirishima during the sampling period.

Species	Abbreviation	Minimum	Median	Maximum	Average ± SD ^a^
PAHs (ng/m^3^)					
Fluoranthene	FR	0.05	0.23	0.72	0.31 ± 0.20
Pyrene	Pyr	0.04	0.14	0.46	0.18 ± 0.11
Benz[*a*]anthracene	BaA	0.01	0.05	0.13	0.06 ± 0.03
Chrysene	Chr	0.02	0.06	0.22	0.09 ± 0.05
Benzo[*b*]fluoranthene	BbF	0.04	0.16	0.48	0.19 ± 0.12
Benzo[*k*]fluoranthene	BkF	0.01	0.06	0.17	0.07 ± 0.04
Benzo[*a*]pyrene	BaP	0.02	0.09	0.19	0.09 ± 0.05
Benzo[*ghi*]perylene	BgPe	0.06	0.18	0.38	0.18 ± 0.09
Indeno[1,2,3-*cd*]pyrene	IDP	0.04	0.15	0.33	0.16 ± 0.08
Total PAHs	ƩPAHs	0.36	1.07	2.90	1.32 ± 0.71
NPAHs (pg/m^3^)					
1-Nitropyrene	1-NP	0.31	1.69	3.87	1.77 ± 1.02
2-Nitropyrene	2-NP	0.14	0.48	0.86	0.46 ± 0.25
2-Nitrofluoranthene	2-NFR	1.53	7.40	17.7	7.75 ± 4.59
Total NPAHs	ƩNPAHs	2.12	9.88	22.3	9.98 ± 5.75
WSIIs (μg/m^3^)					
Sodium	Na^+^	0.03	0.08	0.15	0.07 ± 0.03
Ammonium	NH_4_^+^	0.54	1.30	2.64	1.35 ± 0.59
Potassium	K^+^	0.05	0.09	0.22	0.11 ± 0.06
Calcium	Ca^2+^	0.06	0.10	0.24	0.11 ± 0.04
Magnesium	Mg^2+^	0.01	0.02	0.04	0.02 ± 0.01
Chloride	Cl^−^	<LOD ^b^	0.03	0.10	0.04 ± 0.02
Nitrate	NO_3_^−^	<LOD ^b^	0.19	0.76	0.28 ± 0.23
Sulfate	SO_4_^2−^	1.14	3.49	7.80	3.78 ± 1.77
Total WSIIs	ƩWSIIs	1.96	5.31	11.4	5.74 ± 2.59

^a^: Average ± standard deviation. ^b^: Less than the limit of detection.

**Table 2:** Diagnostic ratios of PAHs, NPAHs, and WSIIs at Kirishima during the sampling period.

	Ratio Values ^a^	Coal Burning	Traffic Emission
[FR]/([FR] + [Pyr])	0.61 (0.50–0.74)	>0.5 ^b^	0.4–0.5 ^b^
[BaA]/[([BaA]/[Chr])	0.41 (0.34–0.52)	>0.35 ^c^	0.2–0.35 ^c^
[BbF]/([BbF] + [BkF])	0.74 (0.72–0.76)	0.78–0.95 ^c^	0.52–0.60 ^c^
[BaP]/[BgPe]	0.57 (0.32–0.90)	>0.6 ^d^	<0.6 ^d^
[IDP]/([IDP] + [BgPe])	0.48 (0.34–0.58)	>0.5 ^e^	0.2–0.5 ^e^
[1-NP]/[Pyr]	0.008 (0.005–0.019)	0.001 ^f^	0.36 ^f^
[NO_3_^−^]/[SO_4_^2−^]	0.11 (0 ^h^–0.32)	<1.0 ^g^	≥1.0 ^g^

^a^: Average (Min–Max) value in this study. ^b^: Rogge et al., 1993 [52]. ^c^: Simcik et al., 1999 [53]. ^d^: Yang et al., 2019 [37]. ^e^: Yunker et al., 2002 [54]. ^f^: Tang et al., 2005 [22]. ^g^: Ye et al., 2017 [43]. ^h^: NO_3_^−^ concentration was <LOD.

**Table 3:** Concentration ratios of WSII species to sodium and at Kirishima during the sampling period.

	Ratio Values ^a^	PM_2.5_^b^	Sea Salt ^b^
[SO_4_^2−^]/[Na^+^]	14.5 (3.28–28.8)	14.2	0.25
[K^+^]/[Na^+^]	1.12 (0.37–4.11)	1.26	0.037
[Ca^2+^]/[Na^+^]	1.08 (0.41–2.27)	1.11	0.038
[Mg^2+^]/[Na^+^]	0.26 (0.10–0.66)	0.54	0.12

^a^: Average (Min–Max) ratio in this study. ^b^: Data referred from Park et al., 2004 [56].

**Table 4:** Benzo[*a*]pyrene-equivalent (BaP_eq_) concentrations (pg/m^3^) at Kirishima during the sampling period.

Species ^a^	TEF ^b^	Minimum	Median	Maximum	Average ± SD ^c^
FR	0.001	0.05	0.23	0.72	0.31 ± 0.20
Pyr	0.001	0.04	0.14	0.46	0.18 ± 0.11
BaA	0.1	1.18	4.80	12.7	5.75 ± 2.88
Chr	0.01	0.16	0.59	2.15	0.87 ± 0.55
BbF	0.1	3.73	15.8	48.0	18.7 ± 12.1
BkF	0.1	1.28	5.65	17.3	6.65 ± 4.28
BaP	1	19.7	85.6	191	90.9 ± 45.8
BgPe	0.01	0.58	1.79	3.80	1.82 ± 0.95
IDP	0.1	4.14	15.0	33.4	16.3 ± 8.11
1-NP	0.1	0.03	0.17	0.39	0.18 ± 0.10
2-NP	- ^d^				
2-NFR	0.05	0.08	0.37	0.88	0.39 ± 0.23
ƩBaP_eq_		31.2	135	302	142 ± 73.8

^a^: Full name are shown in Table 1. ^b^: toxicity equivalent factor (PAHs referred from Nisbet and Lagoy, 1992 [8]; 2-NFR referred from Durant et al., 1996 [9]; 1-NP referred from OEHHA, 2005 [32]). ^c^: Average ± standard deviation. ^d^: No TEF value of 2-NP.

## Data Availability

The data presented in this study are available in the Supplementary material.

## Supplementary Materials

The following are available online at https://www.mdpi.com/1660-4601/18/2/688/s1, Table S1: Limit of detection (LOD) of PAHs, NPAHs, and WSIIs. Table S2: Meteorological conditions at Kirishima during the sampling period. Table S3: Daily concentrations of each PAH (pg/m^3^) and NPAH (pg/m^3^) at Kirishima during the sampling period. Table S4: Daily concentrations of each WSII (µg/m^3^) at Kirishima during the sampling period. Table S5: Main source areas and height ranges of air masses calculated by 72-h tracking backward trajectory arrived at Kirishima during the sampling period.

## References

[1] WHO, Health Effects of Particulate Matter (2013). Policy Implications for Countries in Eastern Europe. Caucasus and Central Asia.

[2] Kim K.-H., Kabir E., Kabir S. (2015). A review on the human health impact of airborne particulate matter. Environ. Int..

[3] Zhang L.L., Zhang X., Xing W.L., Zhou Q.Y., Yang L., Nakatsubo R., Wei Y.J., Bi J.R., Shima M., Toriba A. (2020). Natural aeolian dust particles have no substantial effect on atmospheric polycyclic aromatic hydrocarbons (PAHs): A laboratory study based on naphthalene. Environ. Pollut..

[4] Martinelli N., Olivieri O., Girelli D. (2013). Air particulate matter and cardiovascular disease: A narrative review. Eur. J. Intern. Med..

[5] Zhang H., Zhang L.L., Yang L., Zhou Q.Y., Zhang X., Xing W.L., Kazuichi H., Toriba A., Tang N. (2020). Impact of COVID-19 outbreak on the long-range transport of common air pollutants in KUWAMS. Chem. Pharm. Bull..

[6] Yang Y., Ruan Z., Wang X., Yang Y., Mason T.G., Lin H., Tian L. (2019). Short-term and long-term exposures to fine particulate matter constituents and health: A systematic review and meta-analysis. Environ. Pollut..

[7] Mukherjee A., Agrawal M. (2017). World air particulate matter: Sources, distribution and health effects. Environ. Chem. Lett..

[8] Nisbet I.C., Lagoy P.K. (1992). Toxic equivalency factors (TEFs) for polycyclic aromatic hydrocarbons (PAHs). Regul. Toxicol. Pharmacol..

[9] Durant J.L., Busby W.F., Lafleur A.L., Penman B.W., Crespi C.L. (1996). Human cell mutagenicity of oxygenated, nitrated and unsubstituted polycyclic aromatic hydrocarbons associated with urban aerosols. Mutat. Res..

[10] Taga R., Tang N., Hattori T., Tamura K., Sakai S., Toriba A., Kizu R., Hayakawa K. (2005). Direct-acting mutagenicity of extracts of coal burning-derived particulates and contribution of nitropolycyclic aromatic hydrocarbons. Mutat. Res..

[11] IARC (2013). Bitumens and bitumen emissions, and some n- and s-heterocyclic polycyclic aromatic hydrocarbons. Monographs on the Evaluation of Carcinogenic Risks to Humans Volume.

[12] IARC (2013). Diesel and gasoline engine exhausts and some nitroarenes. Monographs on the Evaluation of Carcinogenic Risks to Humans Volume.

[13] IARC (2015). Outdoor Air Pollution. Monographs on the Evaluation of Carcinogenic Risks to Humans.

[14] Anyenda E.O., Higashi T., Kambayashi Y., Thao N.T.T., Michigami Y., Fujimura M., Hara J., Tsujiguchi H., Kitaoka M., Asakura H. (2016). Exposure to daily ambient particulate polycyclic aromatic hydrocarbons and cough occurrence in adult chronic cough patients: A longitudinal study. Atmos. Environ..

[15] Zhou Q.Y., Zhang L.L., Yang L., Zhang X., Xing W.L., Hu M., Chen B., Han C., Toriba A., Hayakawa K. (2021). Long-term variability of inorganic ions in TSP at a remote background site in Japan (Wajima) from 2005 to 2015. Chemosphere.

[16] Zhou J., Xing Z., Deng J., Du K. (2016). Characterizing and sourcing ambient PM_2.5_ over key emission regions in China Ⅰ: Water-soluble ions and carbonaceous fractions. Atmos. Environ..

[17] Tian M., Wang H., Chen Y., Yang F., Zhang X., Zou Q., Zhang R., Ma Y., He K. (2016). Characteristics of aerosol pollution during heavy haze events in Suzhou, China. Atmos. Chem. Phys..

[18] Zhai G., Zhang N., Dong J., Wang S., Shang K. (2016). Analysis of Association Rules between Hourly Meteorological Factors and PM_2.5_ Water-Soluble Inorganic Ions in Lanzhou, China. Recent Pat. Comput. Sci..

[19] Zhang L.L., Yang L., Zhou Q.Y., Zhang X., Xing W.L., Wei Y., Hu M., Zhao L., Toriba A., Hayakawa K. (2020). Size distribution of particulate polycyclic aromatic hydrocarbons in fresh combustion smoke and ambient air: A review. J. Environ. Sci..

[20] Harrison R.M., Smith D., Luhana L. (1996). Source apportionment of atmospheric polycyclic aromatic hydrocarbons collected from an urban location in Birmingham, UK. Environ. Sci. Technol..

[21] Tang N., Hattori T., Taga R., Igarashi K., Yang X., Tamura K., Kakimoto H., Mishukov V.F., Toriba A., Kizu R. (2005). Polycyclic aromatic hydrocarbons and nitropolycyclic aromatic hydrocarbons in urban air particulates and their relationship to emission sources in the Pan–Japan sea countries. Atmos. Environ..

[22] Arey J., Zielinska B., Atkinson R., Winer A.M., Ramdahl T., Pitts J.N. (1986). The formation of nitro-PAH from the gas-phase reactions of fluoranthene and pyrene with the oh radical in the presence of NOx. Atmos. Environ..

[23] Wang H., Zhu B., Shen L., Xu H., An J., Xue G., Cao J. (2015). Water-soluble ions in atmospheric aerosols measured in five sites in the Yangtze River delta, China: Size-fractionated, seasonal variations and sources. Atmos. Environ..

[24] Zhang J., Tong L., Huang Z., Zhang H., He M., Dai X., Zheng J., Xiao H. (2018). Seasonal variation and size distributions of water-soluble inorganic ions and carbonaceous aerosols at a coastal site in Ningbo, China. Sci. Total Environ..

[25] Poulidis A.P., Takemi T., Shimizu A., Iguchi M., Jenkins S.F. (2018). Statistical analysis of dispersal and deposition patterns of volcanic emissions from Mt. Sakurajima, Japan. Atmos. Environ..

[26] Yang L., Tang N., Matsuki A., Takami A., Hatakeyama S., Kaneyasu N., Nagato E.G., Sato K., Yoshino A., Hayakawa K. (2018). A comparison of particulate-bound polycyclic aromatic hydrocarbons long-range transported from the Asian continent to the Noto Peninsula and Fukue Island, Japan. Asian J. Atmos. Environ..

[27] Yang L., Zhang L.L., Zhang H., Zhou Q.Y., Zhang X., Xing W.L., Takami A., Sato K., Shimizu A., Yoshino A. (2020). Comparative analysis of PM_2.5_-bound polycyclic aromatic hydrocarbons (PAHs), nitro-pahs (NPAHs) and water-soluble inorganic ions (WSIIs) at two background sites in Japan. Int. J. Environ. Res. Public Health.

[28] Shinohara H. (2013). Composition of volcanic gases emitted during repeating Vulcanian eruption stage of Shinmoedake, Kirishima volcano, Japan. Earth Planets Space.

[29] Zhang L.L., Tokuda T., Yang L., Zhou Q.Y., Zhang X., Xing W.L., Wu Q., Zhou Z., Chen R., Kameda T. (2019). Characteristics and health risks of particulate polycyclic aromatic hydrocarbons and nitro-polycyclic aromatic hydrocarbons at urban and suburban elementary schools in Shanghai, China. Asian J. Atmos. Environ..

[30] Yang L., Zhang X., Xing W.L., Zhou Q.Y., Zhang L.L., Wu Q., Zhou Z., Chen R., Toriba A., Hayakawa K. (2021). Yearly variation in characteristics and health risk of polycyclic aromatic hydrocarbons and nitro-pahs in urban shanghai from 2010 to 2018. J. Environ. Sci..

[31] Farren N.J., Dunmore R.E., Mead M.I., Nadzir M., Shahrul M., Samah A.A., Phang S.-M., Bandy B.J., Sturges W.T., Hamilton J.F. (2019). Chemical characterisation of water-soluble ions in atmospheric particulate matter on the east coast of peninsular Malaysia. Atmos. Chem. Phys..

[32] OEHHA (2005). Air Toxics Hot Spots Program Risk Assessment Guidelines.

[33] WHO (2000). Air Quality Guidelines for Europe.

[34] Hayakawa K., Tang N., Nagato E.G., Toriba A., Sakai S., Kano F., Goto S., Endo O., Arashidani K.-i., Kakimoto H. (2018). Long term trends in atmospheric concentrations of polycyclic aromatic hydrocarbons and nitropolycyclic aromatic hydrocarbons: A study of Japanese cities from 1997 to 2014. Environ. Pollut..

[35] Xing W.L., Zhang L.L., Yang L., Zhou Q.Y., Zhang X., Toriba A., Hayakawa K., Tang N. (2020). Characteristics of PM_2.5_-bound polycyclic aromatic hydrocarbons and nitro-polycyclic aromatic hydrocarbons at a roadside air pollution monitoring station in Kanazawa, Japan. Int. J. Environ. Res. Public Health.

[36] Zhang L.L., Morisaki H., Wei Y., Li Z., Yang L., Zhou Q.Y., Zhang X., Xing W.L., Hu M., Shima M. (2020). PM_2.5_-bound polycyclic aromatic hydrocarbons and nitro-polycyclic aromatic hydrocarbons inside and outside a primary school classroom in Beijing: Concentration, composition, and inhalation cancer risk. Sci. Total Environ..

[37] Yang L., Suzuki G., Zhang L.L., Zhou Q.Y., Zhang X., Xing W.L., Shima M., Yoda Y., Nakatsubo R., Hiraki T. (2019). The characteristics of polycyclic aromatic hydrocarbons in different emission source areas in Shenyang, China. Int. J. Environ. Res. Public Health.

[38] Byambaa B., Yang L., Matsuki A., Nagato E.G., Gankhuyag K., Chuluunpurev B., Banzragch L., Chonokhuu S., Tang N., Hayakawa K. (2019). Sources and characteristics of polycyclic aromatic hydrocarbons in ambient total suspended particles in Ulaanbaatar city, Mongolia. Int. J. Environ. Res. Public Health.

[39] Huy D.H., Hien T.T., Takenaka N. (2020). Comparative study on water-soluble inorganic ions in PM_2.5_ from two distinct climate regions and air quality. J. Environ. Sci..

[40] Khan M.F., Hirano K., Masunaga S. (2010). Quantifying the sources of hazardous elements of suspended particulate matter aerosol collected in Yokohama, Japan. Atmos. Environ..

[41] Jiang N., Duan S., Yu X., Zhang R., Wang K. (2018). Comparative major components and health risks of toxic elements and polycyclic aromatic hydrocarbons of PM_2.5_ in winter and summer in Zhengzhou: Based on three-year data. Atmos. Res..

[42] Ye Z., Liu J., Gu A., Feng F., Liu Y., Bi C., Xu J., Li L., Chen H., Chen Y. (2017). Chemical characterization of fine particulate matter in Changzhou, China, and source apportionment with offline aerosol mass spectrometry. Atmos. Chem. Phys..

[43] Nirmalkar J., Batmunkh T., Jung J. (2020). An optimized tracer-based approach for estimating organic carbon emissions from biomass burning in Ulaanbaatar, Mongolia. Atmos. Chem. Phys..

[44] Zhang L.L., Yang L., Zhang H., Zhou Q.Y., Zhang X., Xing W.L., Toriba A., Hayakawa K., Tang N. (2020). Impact of the COVID-19 outbreak on the long-range transport of particulate PAHs in East Asia. Aerosol Air Qual. Res..

[45] Zhang X., Zhang L.L., Yang L., Zhou Q.Y., Xing W.L., Toriba A., Hayakawa K., Wei Y., Tang N. (2020). Characteristics of polycyclic aromatic hydrocarbons (PAHs) and common air pollutants at Wajima, a remote background site in japan. Int. J. Environ. Res. Public Health.

[46] Ma W.-L., Liu L.-Y., Jia H.-L., Yang M., Li Y.-F. (2018). PAHs in Chinese atmosphere part i: Concentration, source and temperature dependence. Atmos. Environ..

[47] Yamasaki H., Kuwata K., Miyamoto H. (1982). Effects of ambient temperature on aspects of airborne polycyclic aromatic hydrocarbons. Environ. Sci. Technol..

[48] Tang N., Sato K., Tokuda T., Tatematsu M., Hama H., Suematsu C., Kameda T., Toriba A., Hayakawa K. (2014). Factors affecting atmospheric 1-, 2-nitropyrenes and 2-nitrofluoranthene in winter at Noto Peninsula, a remote background site, Japan. Chemosphere.

[49] Liu D., Lin T., Syed J.H., Cheng Z., Xu Y., Li K., Zhang G., Li J. (2017). Concentration, source identification, and exposure risk assessment of pm2. 5-bound parent PAHs and nitro-PAHs in atmosphere from typical Chinese cities. Sci. Rep..

[50] Zhang L.L., Morisaki H., Wei Y., Li Z., Yang L., Zhou Q.Y., Zhang X., Xing W.L., Hu M., Shima M. (2019). Characteristics of air pollutants inside and outside a primary school classroom in Beijing and respiratory health impact on children. Environ. Pollut..

[51] Zhang F., Xu L., Chen J., Chen X., Niu Z., Lei T., Li C., Zhao J. (2013). Chemical characteristics of PM_2.5_ during haze episodes in the urban of Fuzhou, China. Particuology.

[52] Rogge W.F., Hildemann L.M., Mazurek M.A., Cass G.R., Simoneit B.R. (1993). Sources of fine organic aerosol. 2. Noncatalyst and catalyst-equipped automobiles and heavy-duty diesel trucks. Environ. Sci. Technol..

[53] Simcik M.F., Eisenreich S.J., Lioy P.J. (1999). Source apportionment and source/sink relationships of pahs in the coastal atmosphere of Chicago and lake Michigan. Atmos. Environ..

[54] Yunker M.B., Macdonald R.W., Vingarzan R., Mitchell R.H., Goyette D., Sylvestre S. (2002). PAHs in the Fraser river basin: A critical appraisal of PAH ratios as indicators of PAH source and composition. Org. Geochem..

[55] Bamford H.A., Baker J.E. (2003). Nitro-polycyclic aromatic hydrocarbon concentrations and sources in urban and suburban atmospheres of the mid-Atlantic region. Atmos. Environ..

[56] Park S., Song C., Kim M., Kwon S., Lee K. (2004). Study on size distribution of total aerosol and water-soluble ions during an Asian dust storm event at Jeju island, Korea. Environ. Monit. Assess..

[57] Tang N., Hakamata M., Sato K., Okada Y., Yang X., Tatematsu M., Toriba A., Kameda T., Hayakawa K. (2015). Atmospheric behaviors of polycyclic aromatic hydrocarbons at a Japanese remote background site, Noto Peninsula, from 2004 to 2014. Atmos. Environ..

[58] Sato K., Takami A., Irei S., Miyoshi T., Ogawa Y., Yoshino A., Nakayama H., Maeda M., Hatakeyama S., Hara K. (2013). Transported and local organic aerosols over Fukuoka, Japan. Aerosol Air Qual. Res..

[59] Kakimoto H., Kitamura M., Matsumoto Y., Sakai S., Kanoh F., Murahashi T., Akutsu K., Kizu R., Hayakawa K. (2000). Comparison of atmospheric polycyclic aromatic hydrocarbons and nitropolycyclic aromatic hydrocarbons in Kanazawa, Sapporo and Tokyo. J. Health Sci..

[60] Hong W.-J., Jia H., Ma W.-L., Sinha R.K., Moon H.-B., Nakata H., Minh N.H., Chi K.H., Li W.-L., Kannan K. (2016). Distribution, fate, inhalation exposure and lung cancer risk of atmospheric polycyclic aromatic hydrocarbons in some Asian countries. Environ. Sci. Technol..

