# Supplementary material for: Atmospheric Behaviour of Polycyclic and Nitro-Polycyclic Aromatic Hydrocarbons and Water-Soluble Inorganic Ions in Winter in Kirishima, a Typical Japanese Commercial City
Source: Int J Environ Res Public Health. 2021 Jan 14;18(2):688. doi: 10.3390/ijerph18020688 (PMC7830530; doi:10.3390/ijerph18020688)
Supplement: Supplementary file 1 [file ijerph-18-00688-s001.pdf]

International Journal of
Environmental Research and Public Health

MDPI

Article

Atmospheric Behaviour of Polycyclic and Nitro-Polycyclic Aromatic Hydrocarbons and Water-Soluble Inorganic Ions in Winter in Kirishima, a Typical Japanese Commercial City

Supplementary Materials contents:

Table S1. Limit of detection (LOD) of PAHs, NPAHs, and WSIIs.

Table S2. Meteorological conditions at Kirishima during the sampling period.

Table S3: Daily concentrations of each PAH (pg/m^3) and NPAH (pg/m^3) at Kirishima during the sampling period.

Table S4: Daily concentrations of each WSII (μg/m^3) at Kirishima during the sampling period.

Table S5: Main source areas and height ranges of air masses calculated by 72-h tracking backward trajectory arrived at Kirishima during the sampling period.

Table S1. Limit of detection (LOD) of PAHs, NPAHs, and WSIIs.

	LOD (pg/mL)		LOD (μg/mL)
FR	16.2	Na^+	0.28
Pyr	30.3	NH_4^+	0.14
BaA	9.1	K^+	0.43
Chr	34.3	Ca^{2+}	0.13
BbF	55.5	Mg^{2+}	0.30
BkF	8.8	Cl^-	0.23
BaP	8.8	NO_3^-	0.08
BgPe	55.3	SO_4^{2-}	0.02
IDP	82.9		
1-NP	12.4		
2-NP	6.2		
2-NFR	5.0		

Table S2. Meteorological conditions at Kirishima during the sampling period.

	T [a]	P [b]	RH [c]	SH [d]	WS [e]	PWD [f]
2016/11/24	11.4	4.5	70	3.4	4.0	NNW
2016/11/25	10.2	0	63	8.6	3.8	NNW
2016/11/26	14.4	0.5	77	5.6	2.7	NNW
2016/11/27	16.6	29.5	93	0	2.7	NNW
2016/11/28	12.3	0	63	7.2	3.7	NNW
2016/11/29	11.5	0	65	9.7	3.0	NNW
2016/11/30	15.4	2	73	5.5	2.2	N
2016/12/1	15.5	0.5	77	4.2	3.2	NNW
2016/12/3	14.0	0	78	7.9	2.7	N
2016/12/5	15.3	0	79	4.5	2.6	NW
2016/12/6	13.4	0	59	7.6	4.3	NNW
2016/12/7	10.7	0	67	9.2	3.7	NNW
2016/12/8	11.7	0	70	6.9	2.6	NNW
2016/12/9	13.3	0	72	7	3.7	NW
2016/12/12	14.1	0	68	1.9	2.4	NNE
2016/12/13	15.8	46.5	94	0	3.2	NNE
2016/12/14	12.4	0	73	4.9	4.2	NW
2016/12/15	9.7	0	67	4.9	4.6	NW
2016/12/16	6.9	0	59	5.9	4.5	NNW
2016/12/18	10.3	0	76	9.0	3.0	NNW
2016/12/19	14.1	0	79	6.7	2.9	N
2016/12/20	16.4	0	85	5.4	2.0	N
Average	13.0	3.8	73	5.7	3.3	

[a]: temperature (°C).

[b]: precipitation (mm).

[c]: relative humidity (%).

[d]: sunshine hour (h).

[e]: average wind speed (m/s).

[f]: prevailing wind direction.

Table S3. Daily concentrations of each PAH (pg/m^3) and NPAH (pg/m^3) at Kirishima during the sampling period.

	FR	Pyr	BaA	Chr	BbF	BkF	BaP	BgPe	IDP	∑PAHs	2-NFR	2-NP	1-NP	∑NPAHs
11/24	216	106	38.9	48.8	74.3	25.9	53.0	105	54.0	722	4.18	0.22	0.85	5.25
11/25	488	192	69.4	104	160	54.7	109	230	155	1561	8.67	0.47	1.59	10.7
11/26	107	43.4	11.8	21.9	37.3	12.8	19.7	68.2	41.4	364	1.53	0.19	0.41	2.12
11/27	606	266	73.2	141	220	70.2	103	219	138	1837	12.2	0.49	2.92	15.6
11/28	667	356	96.9	181	305	103	136	380	229	2453	14.6	0.75	3.56	19.0
11/29	498	181	57.2	96.9	179	65.6	96.0	325	170	1668	8.82	0.62	1.91	11.3
11/30	286	99.0	41.2	54.1	105	37.2	61.7	175	118	977	4.43	0.58	1.04	6.05
12/1	716	457	116	215	477	173	191	277	277	2898	17.7	0.73	3.87	22.3
12/3	154	113	37.8	55.1	120	45.7	65.3	106.0	95.0	791	2.90	0.17	0.83	3.90
12/5	535	379	127	184	480	159	185	341	334	2723	15.7	0.57	3.28	19.5
12/6	322	251	73.9	112	189	74.1	88.2	150	144	1405	7.21	0.26	1.79	9.25
12/7	242	181	55.3	93.7	157	58.4	72.6	124	123	1107	6.85	0.21	1.52	8.59
12/8	363	230	69.5	102	299	115.9	136	263	246	1826	9.89	0.59	2.32	12.8
12/9	367	240	75.7	132	323	122	150	230	298	1937	13.7	0.68	2.35	16.8
12/12	48.4	48.9	13.1	15.9	54.6	18.1	29.3	70.5	83.4	382	2.37	0.21	0.31	2.89
12/13	171	123	43.7	54.0	107	35.8	55.0	67.2	85.3	742	5.30	0.17	0.81	6.28
12/14	225	151	46.8	59.4	116	42.6	58.0	79.3	106	883	3.58	0.16	0.91	4.65
12/15	183	124	35.9	48.3	94.3	31.2	41.1	57.7	78.9	694	3.51	0.14	0.89	4.53
12/16	169	121	45.0	58.3	169	62.4	82.5	145	178	1029	3.90	0.41	1.09	5.41
12/18	106	96.6	43.1	39.5	154	51.8	88.4	206	221	1008	7.95	0.84	2.22	11.0
12/19	112	95.6	44.1	40.6	140	46.9	83.0	182	201	945	8.06	0.86	2.25	11.2
12/20	154	118	49.1	58.9	164	58.4	95.6	200	216	1114	7.59	0.81	2.12	10.5

Table S4. Daily concentrations of each WSII (μg/m³) at Kirishima during the sampling period.

	Na^+	NH_4^+	K^+	Mg^{2+}	Ca^{2+}	Cl^-	NO_3^-	SO_4^{2-}	∑WSIIs
11/24	0.093	0.543	0.058	0.019	0.128	0.038	0.181	1.272	2.330
11/25	0.081	1.234	0.097	0.015	0.100	0.028	0.195	2.866	4.615
11/26	0.092	1.178	0.082	0.010	0.065	0.044	0.090	2.752	4.314
11/27	0.115	1.616	0.130	0.026	0.116	0.036	0.201	4.039	6.277
11/28	0.105	1.910	0.108	0.027	0.239	0.025	0.520	6.433	9.367
11/29	0.077	1.520	0.081	0.024	0.130	< LOD	< LOD	4.580	6.413
11/30	0.090	1.004	0.091	0.013	0.077	0.030	0.645	3.086	5.037
12/1	0.146	2.459	0.211	0.038	0.140	0.030	0.579	7.800	11.403
12/3	0.073	1.604	0.097	0.011	0.061	0.008	0.063	4.613	6.529
12/5	0.091	2.317	0.222	0.029	0.189	0.045	0.733	6.269	9.896
12/6	0.031	1.335	0.213	0.021	0.118	0.021	0.263	3.484	5.487
12/7	0.030	1.418	0.167	0.012	0.118	0.018	0.194	3.582	5.538
12/8	0.060	2.645	0.195	0.022	0.151	0.054	0.759	7.066	10.952
12/9	0.091	1.119	0.134	0.018	0.099	0.019	0.317	2.958	4.754
12/12	0.030	0.555	0.046	0.009	0.069	0.016	0.097	1.136	1.958
12/13	0.067	0.757	0.079	0.026	0.099	0.100	0.167	2.683	3.977
12/14	0.071	0.893	0.080	0.019	0.078	0.023	0.128	2.636	3.927
12/15	0.078	0.739	0.057	0.022	0.085	0.064	0.156	2.386	3.587
12/16	0.034	0.641	0.054	0.010	0.101	0.021	0.122	2.011	2.994
12/18	0.034	1.273	0.087	0.011	0.088	0.028	0.110	3.493	5.125
12/19	0.045	1.382	0.071	0.009	0.105	< LOD	< LOD	3.886	5.498
12/20	0.044	1.489	0.098	0.012	0.131	0.060	0.164	4.207	6.205

Table S5. Main source areas and height ranges of air masses calculated by 72-h tracking backward trajectory arrived at Kirishima during the sampling period.

	Cluster	Main source areas	Main heights
11/24	2	Russia, Mongolia, North China, South Korea, West Japan	500 - 2000 m
11/25	3	Russia, Mongolia, North China, Yellow Sea, West Japan	500 - 5000 m
11/26	1	West Japan, Pacific Ocean	< 500 m
11/27	4	Mongolia, North China, South Korea, West Japan	500 - 2000 m
11/28	3	Russia, Mongolia, North China, Yellow Sea, South Korea, West Japan	500 - 4500 m
11/29	1	South Korea, Sea of Japan, West Japan	< 500 m
11/30	4	Yellow Sea	500 - 1500 m
12/1	3	Russia, Mongolia, North China, Yellow Sea, South Korea, West Japan	500 - 4500 m
12/3	4	Yellow Sea, West Japan	500 - 2500 m
12/5	2	Mongolia, North China, Yellow Sea, South Korea, West Japan	500 - 3500 m
12/6	2	Russia, Mongolia, North China, Yellow Sea	500 - 4000 m
12/7	4	North China, Yellow Sea	500 - 2000 m
12/8	4	South China, Yellow Sea	500 m
12/9	2	Mongolia, North China, Yellow Sea, West Japan	500 - 3500 m
12/12	1	West Japan, Pacific Ocean	< 500 m
12/13	3	Mongolia, North China, Yellow Sea, South Korea, West Japan	500 - 4000 m
12/14	2	Russia, Northeast China, South Korea, West Japan	500 - 5000 m
12/15	2	Russia, Northeast China, South Korea, West Japan	500 - 3500 m
12/16	2	Russia, Northeast China, South Korea, West Japan	500 - 3000 m
12/18	1	South Korea, Sea of Japan, West Japan	< 500 m
12/19	1	Pacific Ocean	< 500 m
12/20	1	Pacific Ocean	< 500 m
